# Pratique de la prophylaxie de la maladie thromboembolique veineuse: enquête réalisée auprès des professionnels de santé de la ville de Ouagadougou

**DOI:** 10.11604/pamj.2014.19.395.5474

**Published:** 2014-12-19

**Authors:** Dangwe Temoua Naibe, André Samadoulougou, Hervé Kabore, Relwendé Aristide Yameogo, Georges Millogo, Nobila Valentin Yameogo, Jonas Koudougou Kologo, Boubacar Jean Yves Toguyeni, Patrice Zabsonre

**Affiliations:** 1 Service de Cardiologie du CHU Yalgado Ouedraogo, Ouagadougou, Burkina Faso; 2 Unité de Formation et de Recherche en Science de la Santé/Université de Ouagadougou, Ouagadougou, Burkina Faso

**Keywords:** MTEV, prophylaxie, professionnel de santé, Ouagadougou, Burkina Faso, VTE, prophylaxy, health professional, Ouagadougou, Burkina Faso

## Abstract

**Introduction:**

L'impact clinique et l'incidence de la maladie thromboembolique veineuse ont conduit à établir des recommandations pour la thromboprophylaxie. L'objectif de notre étude était d’évaluer la pratique de cette prophylaxie par les professionnels de santé de Ouagadougou.

**Méthodes:**

Il s'est agi d'une enquête d'intention réalisée dans le mois de mai 2012 au Centre Hospitalier Universitaire Yalgado Ouédraogo et Centre Médical avec Antenne chirurgicale Paul VI. Un questionnaire a été administré auprès des prescripteurs impliqués dans la prophylaxie de la maladie thromboembolique veineuse (MTEV).

**Résultats:**

Une interview a été administrée à 86 professionnels de santé dont 20 attachés de santé en anesthésie-réanimation, 17 chirurgiens, 11 médecins généralistes et 07 gynécologue-obstétriciens. Leur expérience professionnelle était inférieure à cinq ans dans 65% des cas et ils exerçaient dans 70% des cas au CHU-YO. Les héparines de bas poids moléculaire étaient les plus utilisées (81,4%) avec une intention de prescription significativement plus élevée en réanimation et en chirurgie (p<0,05). Dans 65,7% des cas, la prophylaxie était maintenue jusqu'à la mobilisation des patients. Le coût élevé de l'héparinothérapie représentait une raison de la non utilisation de la prophylaxie dans 46,5% des cas.

**Conclusion:**

Nous constatons que la réalisation de la prophylaxie de la MTEV reste insuffisante à Ouagadougou en dépit de l'existence de recommandations précises de bonnes pratiques. Ces résultats suggèrent la nécessité de la formation médicale continue des professionnels de santé, avec l’établissement des recommandations de pratique clinique adaptée à notre niveau de développement.

## Introduction

La maladie thromboembolique veineuse (MTEV) constitue une thématique majeure de santé publique par sa fréquence et sa gravité croissante [1]. De plus, l'incidence élevée des TVP postopératoires en l'absence de prophylaxie, la gravité de leurs complications et les grandes variations dans la pratique de la prophylaxie ont conduit à l’élaboration récente de recommandations pour la pratique clinique sur la stratification du risque et la prévention de la maladie thromboembolique[2, 3]. En effet, l'efficacité de la thromboprophylaxie en prévention primaire de la maladie thromboembolique a été démontrée par de nombreuses études cliniques. Le guide de pratique élaboré périodiquement par l'American College of Chest Physicians (ACCP) est devenu le standard international [3, 4]. L'utilisation appropriée des recommandations de bonnes pratiques pourrait constituer une alternative de choix pour réduire la morbimortalité des MTEV [5, 6]. Une connaissance adaptée des professionnels de santé en rapport avec la MTEV, la disponibilité financière et géographique des ressources thérapeutiques pourraient affecter considérablement les pratiques. De plus, l'usage de la prophylaxie de la MTEV est peu connu en Afrique et il n'existe pas d’études au Burkina Faso évaluant cette pratique. L'objectif de notre travail était d’évaluer la pratique de la prophylaxie de la MTEV auprès des professionnels de santé de la ville de Ouagadougou.

## Méthodes

Il s'est agi d'une enquête d'intention, réalisée durant la période allant du 1^er^ au 31 Mai 2012. Un questionnaire a été administré au cours d'un entretien semi-dirigé par les investigateurs (médecins en spécialisation de cardiologie) et après obtention du consentement, auprès des prescripteurs impliqués dans la prophylaxie de la MTEV. Les professionnels de santé exerçaient au centre hospitalier universitaire Yalgado OUEDRAOGO (CHU-YO) et au centre médical avec antenne chirurgicale (CMA) de Paul VI de la ville de Ouagadougou. Ont été inclus: médecins anesthésiste-réanimateurs, gynécologue-obstétriciens, chirurgiens, médecins généralistes des services des urgences, attachés de santé en anesthésie-réanimation, attachés de santé en chirurgie, les sage-femmes. Etaient renseignés: la qualification et l'ancienneté des prescripteurs; leur connaissance des facteurs de risque de MTEV, des principaux signes et symptômes évocateurs, de même que leur connaissance des procédures diagnostiques; le type de prophylaxie utilisé, ainsi que sa durée; les raisons de la non utilisation de l'héparinotherapie. Les données ont été analysées avec le logiciel EPI INFO version 7. Le test de Khi^2^ a été utilisé comme test statistique. Il était significatif si p<0,05.

## Résultats

**Figure 1:**
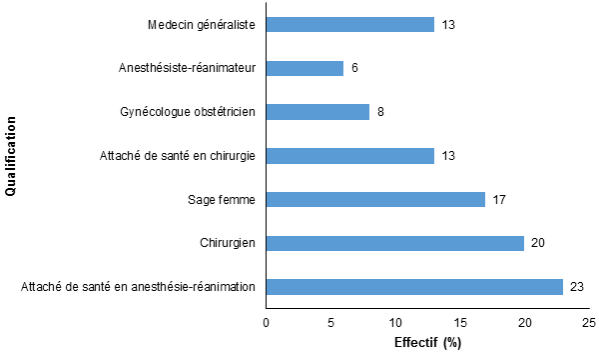
Répartition des prescripteurs selon leur qualification

Nous avons interviewé 86 professionnels de santé dont 20 attachés de santé en anesthésie-réanimation, 17 chirurgiens. La Figure 1 nous donne la répartition des prescripteurs selon leur qualification. L'ancienneté moyenne des prescripteurs était de 6,4 ans ± 6,8 (1 à 25 ans). Leur expérience professionnelle était inférieure à cinq ans dans 65% des cas, entre cinq et 10 ans dans 11,7% des cas et supérieure à 10 ans dans 23,3% des cas. Ils exerçaient dans 70% des cas au CHU-YO.

Indications de prophylaxie des MTEV (connaissance des facteurs de risque): l'immobilisation-plâtrée était noté dans 73,3% des cas. Le Tableau 1 nous donne la répartition des facteurs de risque de MTEV connus par les professionnels de santé. Environ 29,2% des prescripteurs connaissaient au moins 5 facteurs de risque.

**Tableau 1:** Facteurs de risque de MTEV connus par les professionnels de santé

Facteurs de risque	Effectifs	Pourcentage (%)
Immobilisation-plâtrée	63	73,3
Chirurgie majeure	44	51,2
Traumatisme	33	38,4
Néoplasie	07	8,1
ATCD de MTEV	09	10,5
Post partum/grossesse	34	39,5
Thrombophilie	16	18,6
Obésité	53	61,6
Age > 60 ans	17	19,8
Insuffisance cardiaque	28	32,6
Insuffisance veineuse	20	23,3
Maladies infectieuses/inflammatoires	06	7,0
VIH	03	3,5
Tabac	17	20
HTA	08	9,3
Diabète	09	10,5

Indicateurs cliniques de suspicion de TVP/EP (connaissance des principaux signes et symptômes): les lésions cutanées (96,5%) et la douleur (94,2%) étaient rapportées comme signes et symptômes de thrombose veineuse profonde (TVP), alors que pour l'embolie pulmonaire (EP), il s'agissait de la dyspnée (87,2%) et de la douleur thoracique (73,3%). Le Tableau 2 donne les principaux signes et symptômes évocateurs de la TVP et de l'EP.

**Tableau 2:** Connaissance des principaux signes et symptômes évocateurs de TVP et d'EP

Principaux signes et symptômes	Effectifs	Pourcentage(%)
TVP		
Douleur	81	94,2
Œdème	64	74,4
Fièvre	53	61,6
Lésions cutanées	83	96,5
Signe de HOMANS	32	37,2
Varice	07	8,1
Diminution du ballotement du mollet	34	39,5
Impotence fonctionnelle	22	25,6
EP		
Dyspnée	75	87,2
Douleur thoracique	63	73,3
Toux	34	39,5
Tachypnée	16	18,6
Anxiété	20	23,3
Hémoptysie	19	22,1

Procédures diagnostiques possibles (connaissance des moyens complémentaires pour le diagnostic de TVP/EP): l’échographie Doppler veineux (79,1%) et l'angioscanner thoracique (51,2%) étaient cités comme procédures diagnostiques respectivement dans la TVP et l'EP. Le Tableau 3 montre les différentes procédures diagnostiques à considérer en cas de TVP et d'EP.

**Tableau 3:** Connaissance des procédures diagnostiques par les prescripteurs

Techniques	Fréquence	Pourcentage (%)
TVP		
Echographie Doppler veineux	68	79,1
D.dimères	30	34,9
Bilan de la coagulation	23	26,7
EP		
Radiographie thoracique	59	68,6
ECG	30	34,9
Echocardiographie Doppler	06	25,6
D.dimères	33	38,4
Scintigraphie pulmonaire	18	20,9
Angioscanner thoracique	44	51,2
Bilan de la coagulation	22	25,6

### Prophylaxie

**Figure 2:**
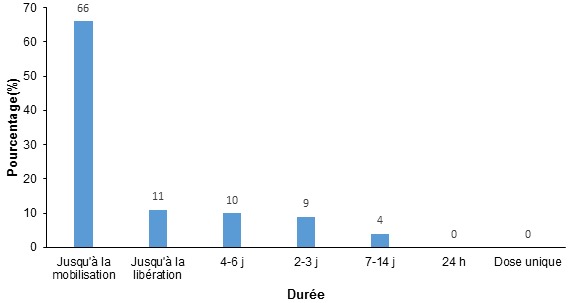
Durée de la prophylaxie envisagée par les professionnels de santé

Environ 80% des professionnels de santé déclaraient utiliser les héparines de bas poids moléculaire pour la prophylaxie des MTEV. Une intention de prescription significativement plus élevée était retrouvée en réanimation et en chirurgie (p = 0,02 et 0,04). Les dispositifs physiques ont été utilisés dans sept pour cent des cas. Quarante sept prescripteurs (54,6%) envisageaient une association HBPM et utilisation des moyens physiques (Tableau 4). Dans 65,7% des cas, la prophylaxie était maintenue jusqu’à la mobilisation des patients (Figure 2). Le coût élevé de l'héparinothérapie représentait une raison de la non utilisation de la prophylaxie dans 46,5% des cas (Tableau 5).

**Tableau 4:** Types de prévention utilisés par les professionnels de santé

Type de technique	Effectifs	Pourcentage(%)
HBPM	70	81,4
AVK	19	22,1
Antiagrégant plaquettaire	24	28,0
Mobilisation précoce	55	64,0
Compression/contention élastique	06	7,0

**Tableau 5:** Raisons de la non-utilisation de la prophylaxie

Raisons	Effectifs	Pourcentage (%)
Faible incidence présumée	17	20,0
Risque hémorragique élevé	25	29,1
Coût élevé de l'héparine	40	46,5
Intolérance à l'héparine	08	9,3

## Discussion

La distribution des professionnels de santé dans notre étude est due à leur répartition et surtout à leur disponibilité dans les différents centres de santé. Le biais de sélection est un souci et peut constituer une limite quant à la généralisation de nos résultats. Les recommandations précises sur le diagnostic et la prophylaxie des MTEV sont disponibles et reposent sur une littérature solide. Leur mise en pratique n'est pas toujours effective. Très peu de résultats sont disponibles sur l’évaluation de l'application de ces recommandations. Une étude internationale récente ENDORSE de 35 329 patients hospitalisés a montré que seulement 58,5% des patients chirurgicaux et 39,5% des patients médicaux recevaient une prophylaxie appropriée [7]. Nos résultats montrent une insuffisance des connaissances des professionnels de santé dont la plupart relève du personnel paramédical (53,5%) à identifier les facteurs de risque, les signes et symptômes évocateurs de MTEV. Une attention particulière est cependant accordée aux signes les plus évocateurs. En Italie, Stefano de Franciscis et al. dans une enquête nationale sur la pratique clinique des recommandations sur la prophylaxie de la MTEV faisaient les mêmes constatations [8]. Ils retrouvaient: pour la TVP: la douleur (89,3%) et l’œdème des membres inferieurs (76,3%), la fièvre (24.4%), l'impotence fonctionnelle (20,6%), la manœuvre de Homans positive (19,8%), un réseau veineux collatéral (14,5%); pour l'EP: dyspnée (92,5%), douleur thoracique (70,1%), tachypnée (50,7%), toux (27,6%), hémoptysie (26,8%), angoisse (19,4%), sueurs (19,4%). Les réponses ont montré une méconnaissance également des algorithmes diagnostiques d'imagerie (Echographie Doppler veineux 79,1%, angioscanner thoracique 51,2%) et de laboratoire (D.dimères 34,9% pour la TVP et 38,4% pour l'EP), permettant de confirmer la MTEV. Ce qui pourrait s'expliquer par l'insuffisance des ressources d'imagerie et de laboratoire nécessaires pour confirmer ce diagnostic dans nos structures hospitalières. Le respect des recommandations passe par au moins deux choses: connaitre les recommandations et identifier les patients à risque. Hors tous les prescripteurs dans notre étude, affirmaient ne pas disposer de protocole de pratique de la thromboprophylaxie, contre 8% des prescripteurs du Togo [9] et 58% en Malaisie [10]. Des efforts sont à faire pour renforcer leur niveau de connaissance. En effet la mise en place de programmes de formation continue des professionnels de santé pourrait constituer un gage certain et permettre d'améliorer leur pratique médicale de façon générale.

Nos résultats indiquent que les HBPM constituent le moyen de prophylaxie le plus utilisé; le taux de prophylaxie envisagée dans notre étude (81,4%) était comparable à celui retrouvé par Ouro-Bang'na[9] au Togo en période post-opératoire (87%). Cette valeur est supérieure à celle de Prasannan[10] en Malaisie (73%) et nettement inférieure à celle de Williams [11] au pays de Galles (100%). Dans ce dernier pays, les praticiens associaient les héparines et les moyens physiques dans 89% des cas contre seulement 33% au Togo et 54,6% dans notre étude. Aux États-Unis, 96% des chirurgiens ayant répondu à une enquête évaluant leur préférence entre HBPM et HNF, faisaient de la prophylaxie de la MTEV; les moyens physiques étaient utilisés par 56% des chirurgiens alors que l'association moyens physiques et moyens pharmacologiques était utilisée par seulement 26% des chirurgiens [12]. Ces pays (Galles et USA) ont de longue date été sensibilisés à l'utilisation des HBPM en prophylaxie médicochirurgicale avec des décisions thérapeutiques très protocolisées. Ce qui expliquerait leurs relatifs bons résultats. Il faudrait tout de même interpréter avec prudence cette intention élevée de prescription des HBPM par les professionnels de santé dans notre contexte. En réalité, il y a une sous prescription de la thromboprophylaxie comme c'est le cas au Togo (8%) contre 83,7% au Québec [13]. Cette constatation pourrait être attribuable à plusieurs facteurs: la méconnaissance des indications de la thromboprophylaxie, la faible incidence présumée de la MTEV, l'incidence élevée du risque hémorragique et le coût élevé de l'héparinotherapie. Une étude observationnelle parait nécessaire pour compléter l’évaluation de cette pratique. Les HBPM constituent actuellement le traitement de référence dans la prévention de la MTEV, en termes d'efficacité, de commodité d'emploi, de réduction de risque hémorragique et de réduction du risque de thrombopénie induite comparés aux HNF [2, 4]. Notons que si dans notre enquête les méthodes physiques étaient essentiellement la mobilisation précoce et la contention/compression élastique, elles étaient plus fournies dans les pays occidentaux et asiatiques avec la compression pneumatique intermittente et bien d'autres méthodes plus onéreuses. Les recommandations bien que précises, adaptées et schématiques pour la thromboprophylaxie sont parfois d'application difficile dans nos sociétés africaines aux possibilités économiques limitées et surtout caractérisées par une insuffisance des possibilités diagnostiques et thérapeutiques des structures de santé. D'où un intérêt à établir des protocoles de pratique adaptés à notre environnement pour un minimum de sécurité de nos patients. Ce qui a été également suggéré par les recommandations de la 8ème conférence de l'ACCP [3].

## Conclusion

Nous constatons que la réalisation de la prophylaxie de la MTEV reste insuffisante à Ouagadougou en dépit de l'existence de recommandations précises de bonnes pratiques. Ces résultats suggèrent la nécessité de la formation médicale continue des professionnels de santé, avec l’établissement des recommandations de pratique clinique adaptée à notre niveau de développement. Ce qui représenterait une vraie possibilité de contrôle de la morbidité et de la mortalité de la MTEV.
